# When the smoke gets in your lungs: short-term effects of Indonesia’s 2015 forest fires on health care use

**DOI:** 10.1186/s12940-024-01079-x

**Published:** 2024-05-03

**Authors:** Novat Pugo Sambodo, Menno Pradhan, Robert Sparrow, Eddy van Doorslaer

**Affiliations:** 1 Erasmus University Rotterdam, Erasmus School of Health Policy & Management, P.O. Box 1738, Rotterdam, 3000 DR The Netherlands; 2 Department of Economics, Faculty of Economics and Business, Universitas Gadjah Mada, Yogyakarta, Indonesia; 3 University of Amsterdam, Amsterdam, The Netherlands; 4 Vrije Universiteit Amsterdam, Amsterdam, The Netherlands; 5 Amsterdam Institute for Global Health & Development, Amsterdam, The Netherlands; 6 Wageningen University, Wageningen, The Netherlands; 7 International Institute of Social Studies, Erasmus University Rotterdam, The Hague, The Netherlands; 8 Australian National University, ACT Canberra, Australia; 9 Erasmus School of Economics, Erasmus University Rotterdam, Rotterdam, The Netherlands

## Abstract

**Background:**

The forest fires that ravaged parts of Indonesia in 2015 were the most severely polluting of this century but little is known about their effects on health care utilization of the affected population. We estimate their short-term impact on visit rates to primary and hospital care with particular focus on visits for specific smoke-related conditions (respiratory disease, acute respiratory tract infection (ARTI) and common cold).

**Method:**

We estimate the short-term impact of the 2015 forest fire on visit rates to primary and hospital care by combining satellite data on Aerosol Optical Depth (AOD) with administrative records from Indonesian National Health Insurance Agency (BPJS Kesehatan) from January 2015–April  2016. The 16 months of panel data cover 203 districts in the islands of Sumatra and Kalimantan before, during and after the forest fires. We use the (more efficient) ANCOVA version adaptation of a fixed effects model to compare the trends in healthcare use of affected districts (with AOD value above 0.75) with control districts (AOD value below 0.75). Considering the higher vulnerability of children’s lungs, we do this separately for children under 5 and the rest of the population adults (> 5), and for both urban and rural areas, and for both the period during and after the forest fires.

**Results:**

We find little effects for adults. For young children we estimate positive effects for care related to respiratory problems in primary health care facilities in urban areas. Hospital care visits in general, on the other hand, are negatively affected in rural areas. We argue that these patterns arise because accessibility of care during fires is more restricted for rural than for urban areas.

**Conclusion:**

The severity of the fires and the absence of positive impact on health care utilization for adults and children in rural areas indicate large missed opportunities for receiving necessary care. This is particularly worrisome for children, whose lungs are most vulnerable to the effects. Our findings underscore the need to ensure ongoing access to medical services during forest fires and emphasize the necessity of catching up with essential care for children after the fires, particularly in rural areas.

**Supplementary Information:**

The online version contains supplementary material available at 10.1186/s12940-024-01079-x.

## Introduction

The forest fires that affected two islands in Indonesia in 2015, between June and October, were the most severe and polluting of this century, producing more CO_2_ emissions than the average daily greenhouse gas emissions in the entire US economy [1, 2]. The fires burned around 2.6 million hectares of land, estimated at four and half times the size of Bali [3]. As a result of this, with around 96.937 active fires detected, global emissions in Indonesia skyrocketed with approximately 1,043 million tons of CO_2_. During the peak months of Sept-Oct, the CO_2_ emissions exceeded that of the entire European Union [4] and endangered the life and health conditions of the Indonesian population. This research estimates the impact of the 2015 fires on health care utilization in primary care facilities and hospitals that participate in the national health insurance scheme. The aim is to investigate to what extent the health system was able to provide the medical respiratory care that is required to mitigate any potential negative health impacts.

Forest fires can have severe long lasting impacts on human health. For Indonesia, these have been studied extensively for the 1997 forest fires, which were even larger than those in 2015, emitting about four times as much CO_2_ [4]. The population census of 2000 recorded 15,600 fewer children than expected in the birth cohort of the affected areas, which [5] was attributed mostly to early childhood mortality. Children that did survive also had lower human development outcomes. They were found to grow shorter in height [6], to have lower lung capacity [7] and to score 6% lower on cognitive tests by age 8 [8]. Adults exposed to the fires were less able to perform daily activities in the year of the forest fire [9].

Appropriate and prompt treatment is crucial to reduce morbidity from wildfire smoke exposure [10]. Children, because they inhale more smoke relative to their body weight and because their functions are still developing, and those with pre-existing respiratory conditions are particularly vulnerable. Most of the evidence on health care utilization in response to forest fires stems from developed countries. In Singapore, for instance, Sheldon and Sankaran [11] found that the Indonesian haze in 2013–2015 increased polyclinic attendances for acute respiratory tract infections and acute conjunctivitis. These visits were linked to the deterioration in air quality during the haze. For Australia, which is also prone to frequent forest fires, Chen et al. [12] reported that bushfire smoke in Brisbane was significantly linked to increased hospital visits for respiratory illness. In America the forest fires in Hoopa Valley, California, caused a particulate matter that led to increasing asthma, coronary artery disease and headache visits of Hoopa and nearby communities [13]. Similar studies in low- and middle income countries are far fewer because air quality stations in forests or land are scarce, and not easily related to reliable administrative data on health records. This study intends to contribute to filling this gap.

For Indonesia, the literature on the health effects of forest fires is relatively small. Arifin and Setyawan [14] compared the health of people living in municipalities with and without the presence of a palm oil company in 2014. Palm oil companies use forest fires to clear land, and they find that communities that host them more often experience forest fires and have worse health outcomes. Our study contributes to this study by focusing on particularly severe forest fire, and we compare control and treatment groups based on the AOD level (related to smoke), rather than the presence of a palm oil plantation. In spite of differences, Arifin and Setyawan [14] do report similar results regarding the impact of forest fires on children’s health (increasing the probability of asthma in children) and on hospitalizations of children (and elderly) for respiratory reasons. Moreover, our study exploits information on healthcare visits for smoke-related diseases on a much more frequent, i.e. monthly basis, which is not provided in the other studies.

Further, Jayachandran [5] investigated the impact of the Indonesian 1997 forest fire on fetal infant and child mortality. She analyzed combined data of particulate measures (UV Aerosol Index) and Indonesian 20,000 census data, and found that the probability of children surviving declined if they resided in a forest fire location during a forest fire time. Our study add to this literature on the relationship between healthcare visits and smoke-related diseases, even though in a different episode (2015 forest fire). Unlike Jayachandran and Arifin, who employed cross-sectional data, we use longitudinal district level data, collected on a monthly basis, and also captured effects after the forest fire period ended.

To investigate whether the effects of forest fire are the result of changes in the need for health care or the accessibility of health care, we estimate impacts both for general outpatient care and for care specifically related to respiratory problems. We would expect the latter to respond most to need effects. We combine data on Aerosol Optical Depth (AOD) obtained from satellite data with administrative data from the National Health Insurance (JKN) members’ utilization of outpatient services in primary health facilities and hospitals, in districts affected by the fires and in a selected set of control districts. The detailed diagnosis codes included in the JKN data allow us to focus our analysis on treatment for respiratory conditions and do a separate analysis for children below five. We consider both the period of the forest fires, and the six months succeeding the forest fires. While in the latter period the smoke had disappeared, residents may still need care because they could not access it during the fires, or because their health condition has not yet improved. We apply an ANCOVA estimation framework that corrects for outcomes observed in the pre-forest fire period.

The remainder of the paper is structured as follows. Section 2 discusses how forest fires could affect health and the required health care responses. Section 3 describes the construction of the analysis dataset, Sect. 4 the estimation method and Sect. 5 presents the results. We discuss the implications of the results in Sect. 6. Finally, Sect. 7 presents the conclusion of this research.

## Forest fire smoke, health, and healthcare use

Forest fire smoke produces a complex mixture of gases, particles, water vapor, organic debris, and minerals due to incomplete combustion. Its characteristics depend on a number of variables, including the kind and amount of materials (wood and plants) that is burned, the temperatures that the fires produce, as well as the wind and weather conditions more generally [15]. Smoke from forest fires typically contains three components: (a) gases like, for example, sulphur dioxide (SO_2_), nitrogen oxides (Nox), carbon monoxide (CO), carbon dioxide (CO_2_), and others. (b) Particulate matter (PM) describes the particles deriving from forest fires classified by their size. Particles sizes larger than 10 micrometers can irritate the eyes, nose, and throat but typically do not penetrate the lungs. Smaller particles can be inhaled into the lungs. Sizes between 2.5 and 10 micrometers are classified as coarse particles (PM10), while sizes of 0.1 to 2.5 micrometers are considered fine particles (PM2.5). (c) Other materials in a somewhat larger quantity, such as metal, dioxin, benzene, toluene, and polycyclic aromatic hydrocarbon (PAH) [15].

The primary contaminant is particulate matter (PM) and its effects on human health depend on particle size. PM10 enters through the throat and nose and deposits in the heart and surface of the lungs. This PM10 deposit can cause inflammation and tissue damage [16]. Further, PM 2.5 can enter the deeper part of the lungs and even can go to bloodstream [15, 17]. Population groups which are more sensitive to smoke from forest fires include: seniors, expectant mothers, children, people who have had heart or lung disease in the past (such as those who have asthma, chronic obstructive pulmonary disease, or COPD), and those who are pregnant. People with other chronic illnesses may also be at greater risk [15].

## Data

**Fig. 1:**
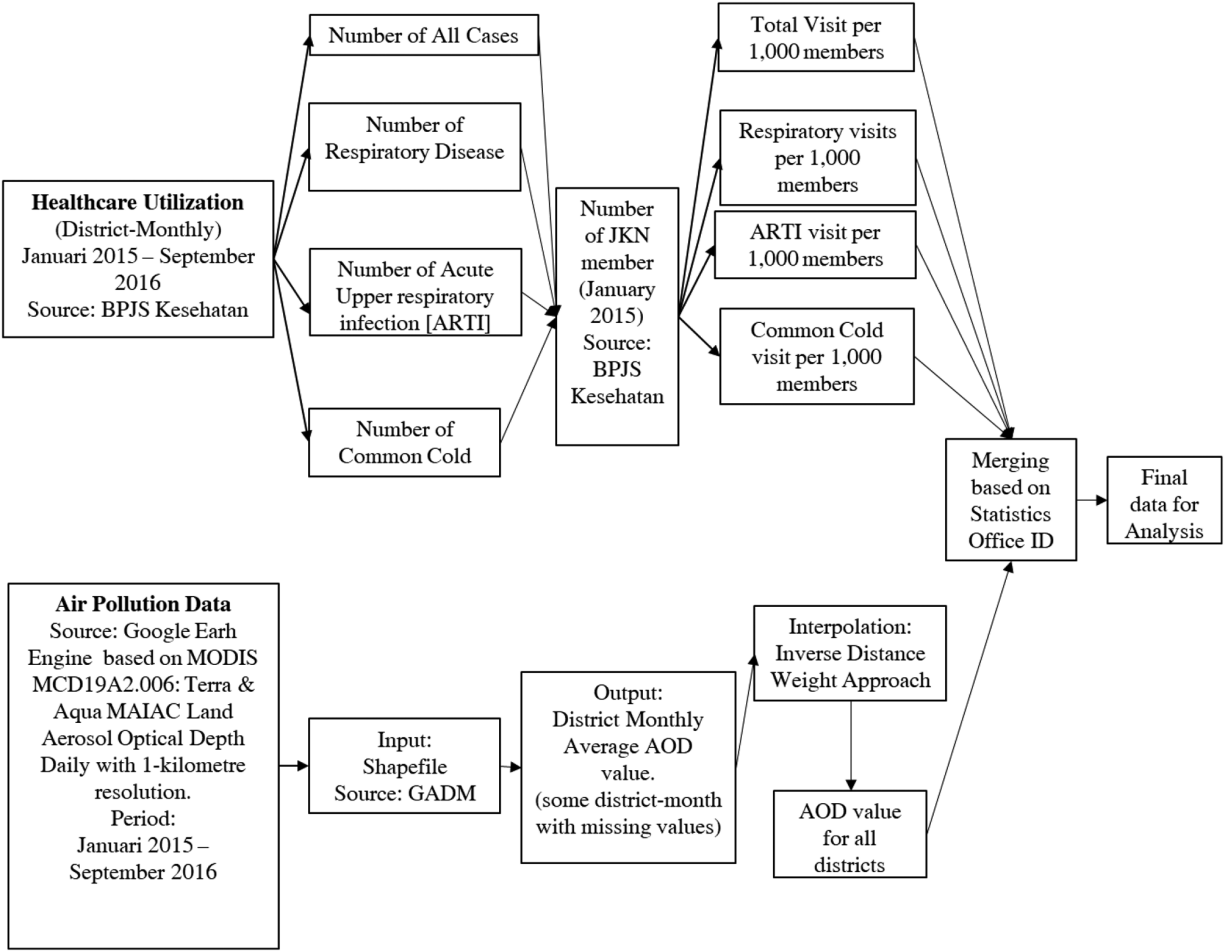
Flowchart of data construction for analysis

BPJS Kesehatan has provided us with monthly district aggregated data of selected ICD10 [18] coded utilization from all JKN members for the purpose of this study. The mean JKN coverage in our district sample is 57%, with 64.08% in city/urban and 52.30% in regency/rural. While JKN coverage is far from complete, we believe that it nonetheless provides a good impression of the utilization responses in the area. We expect that JKN members are more likely to use the care in response to the forest fires as compared to non JKN members. If this is the case, then our estimates are an upper bound of the demand response. Data on fire and air quality were obtained from the MODIS satellite scan. We get shapefile data from the Global Administrative Areas (GADM) database that provides us with a statistical geolocation code [19] that enables us to merge air pollution data and healthcare utilization at the district level (See Fig. 1).

### Healthcare utilization data

Our dependent variables are the monthly district-level aggregates of all outpatients’ visits and outpatient visits for smoke related diseases (respiratory illness, common cold and ARTI) of JKN members to primary care and hospitals. The healthcare utilization data are monthly observations from January 2015 to April 2016 (16 months) for 203 districts located in Sumatra and Kalimantan Islands for 4 aggregates: (i) total outpatient visits, (ii) all type of respiratory disease (ICD10: J00-J22), and the two most common diagnoses related to forest fire smoke namely (iii) Common Cold (Nasopharyngitis) (ICD10: J00) and (iv) Acute Respiratory Tract Infection (ARTI) (ICD10: J069) [20]. The typical symptoms of common cold such as sneezing, nasal congestion, and excess mucus production [21] are also present during smoke related respiratory problems. Meanwhile, ARTI common signs are fever, cough, cold, out of breath, weight loss and myalgia [22]. Because the symptoms for these two diseases tend to occur in the first two weeks after the first contamination these types of utilization are suitable to evaluate short term effects of smoke inhalation.

We obtain visit rates per 1,000 JKN members for each type of utilization by dividing monthly district visits by the number of JKN members in a district (in the respective age group) and multiply by 1,000. We use the number of JKN members on January 2015 as the denominator for all periods to avoid problems with endogenous changes in enrolment in JKN. We construct separate utilization rates for those under five years old and those five years and older, and for rural (Regency) and urban (City) districts.

### Air Pollution data

#### Aerosol Optical depth (AOD)

We use Aerosol Optical Depth (AOD) from satellite imagery as our main measure of smoke pollution following approach from [23]. AOD is closely linked to Particulate Matter 2.5 (PM 2.5) [24] which can be detrimental to human health if its value exceeds a critical level [25]. Aerosol optical depth (AOD) is determined by airborne particles like dust, smoke, and pollution that may obstruct sunlight by either absorbing it or dispersing it. It indicates the amount of direct sunlight that is blocked from reaching the ground by these aerosol particles. One main advantage of AOD is that global coverage is possible by computing spatial averages over the user-selected areas (districts in our case) over a given time period in Google Earth Engine system (GEE) [26]. A higher value of AOD generally indicates a higher share of aerosols like smoke and dust in the atmosphere.

For Indonesia, an AOD value above 0.75 represents a high concentration of aerosols in the atmosphere from biomass burning [27]. Most studies that look at impacts of smoke in developed countries used PM2.5 as a measure of air pollution, which is measured through ground stations. Because this information is not widely available for Indonesia, we use the AOD measure instead. The global maximum AOD that is reported by MODIS sensors is 5 and most of the distribution of AOD values are between 0 and 0.5.

The Google Earth Engine (GEE) database that we use is widely used for public health-environment research^1^. It contains atmospheric data from various satellites. We use MODIS MCD19A2.006: Terra & Aqua MAIAC Land Aerosol Optical Depth Daily with 1-kilometre resolution data within GEE database. GEE produces the AOD value of the district shapefile that we input into the GEE by taking the monthly average of daily data. The district-monthly AOD can then be merged with the district-monthly healthcare utilization using Indonesian Statistics Office (Badan Pusat Statistik) geolocation identification. In case clouds are covering the land when the satellite is crossing, there are missing values that do not contribute to the spatial averages. In such cases we use geospatial interpolation, the details of calculation and assessment of interpolation accuracy are explained in Appendix 1.

## Methods

This study is quantitative research with causal design using longitudinal data. We use data from three different sources. We link primary care and hospital admission data from the Indonesian National Health Insurance Agency (JKN) with geospatial location and air quality data. We define districts as being affected by the forest fire if the AOD level during the 5-month (June-October 2015) period of the forest fire exceeds 0.75, which is a locally validated threshold for unhealthy AOD values [27]. Figure in Appendix 2 presents the average AOD levels in treatment and control district by month. AOD levels peaked in October 2015 at 2.55 in treatment districts.

Our estimation model is based on the general idea that healthcare utilization is a function of our air quality indicator (AOD). We estimate this function separately for children under 5 (because children’s lungs are more vulnerable) and the rest of the population (> 5), for both urban and rural locations. Because Appendix 2 shows that there is a clear spike in AOD values during the forest fire period June-October 2015, we use an ANCOVA specification of the regression model to account differences between affected and non-affected districts before the forest fire period, by including pre-fire outcome${H}_{k,0}$ (averaged over January-May 2015) as control variable. The main advantage of ANCOVA over a conventional difference-in-difference (DID) specification is gains in efficiency resulting in lower estimated standard errors [28]. In terms of identification of causal effects, both methods (DID and ANCOVA) rely on the parallel trend assumption that, in the absence of the forest fires and elevated AOD levels, the trends in health care utilization would have been the same in affected and non-affected districts.

The variable ${Affected}_{k}$ equals 1 if a district *k* experiences an average AOD of more than 0.75 during forest fire period June-October 2015. The dummy variable ${During fire}_{t}$ equals 1 for the months of the forest fire, and the variable ${After fire}_{t }$equals one for the six months after the forest fire, November 2015 – April 2016. Our estimates of interest are the coefficients for the interaction terms of ${Affected}_{k}$ with the two different periods: ${\sigma }_{1}$ captures the impact of fire induced air pollution during the forest fire period, while ${\sigma }_{2}$capture delayed effects that occurred after the period of fire. We also include time dummies ${\alpha }_{t}$. This yields the following ANCOVA specification:

1 $$\eqalign{{H_{k,t}} = & {\sigma _0}\, + \,{\sigma _1}\,\,During\,fir{e_t}*Affecte{d_k}\, \cr & + \,{\sigma _2}After\,fir{e_t}*Affecte{d_k} + {\sigma _3}{H_{k,0}} + {\alpha _t} + {\varepsilon _{kt}} \cr}$$

We also provide standard difference-in-difference (DID) estimates as a robustness test to the ANCOVA results. We expect the DID results to show higher standard errors compared to the ANCOVA.

## Results

Table 1 shows summary statistics of our sample pre-fire, during fire and after fire. We find that all age primary care use in affected districts for total, respiratory disease and common cold is lower than in control districts in pre-fire, during and after fire period (but only for common cold it is statistically significant). In contrast, the visit rate for all outcomes of under five years old are higher but these differences are not statistically significant. For hospital care we see an opposite pattern, with higher utilization in non-affected districts in all time periods. The mean AOD value is about the same during the pre-fire period, with mean AOD of 0.30 and 0.28 for the affected and control districts, respectively. During the forest fire period, the mean AOD levels jump sharply for affected districts to 1.12, and slightly for control districts to 0.52. After the fires, the air quality returns back to pre-fire levels.

**Fig. 2:**
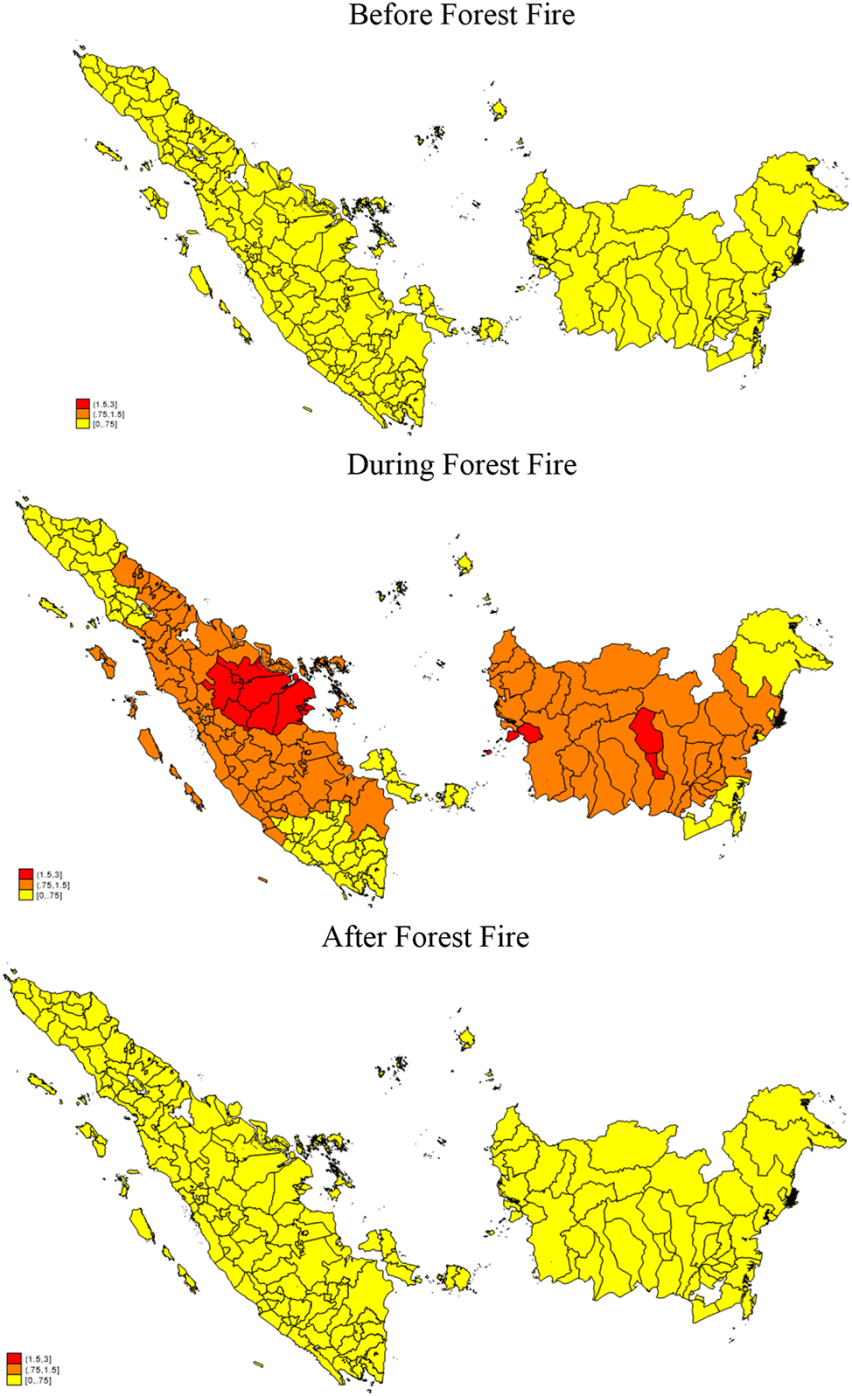
Maximum AOD Level before, during and after forest fire period in Sumatera and Kalimantan. Source: Aerosol Optical Depth derived from MODIS satellite data. Note: Pre-Fire (January-May 2015); During Fire (June-October 2015); After Fire (November 2015 – April 2016)

Figure 2 maps the AOD maximum values before, during and post forest fires. The AOD values are normally below 0.75 represented by light yellow for all districts in Sumatera and Kalimantan in before and after forest fire period. This is in line with a similar spike in the national AOD levels for the same period observed by [29].

**Fig. 3:**
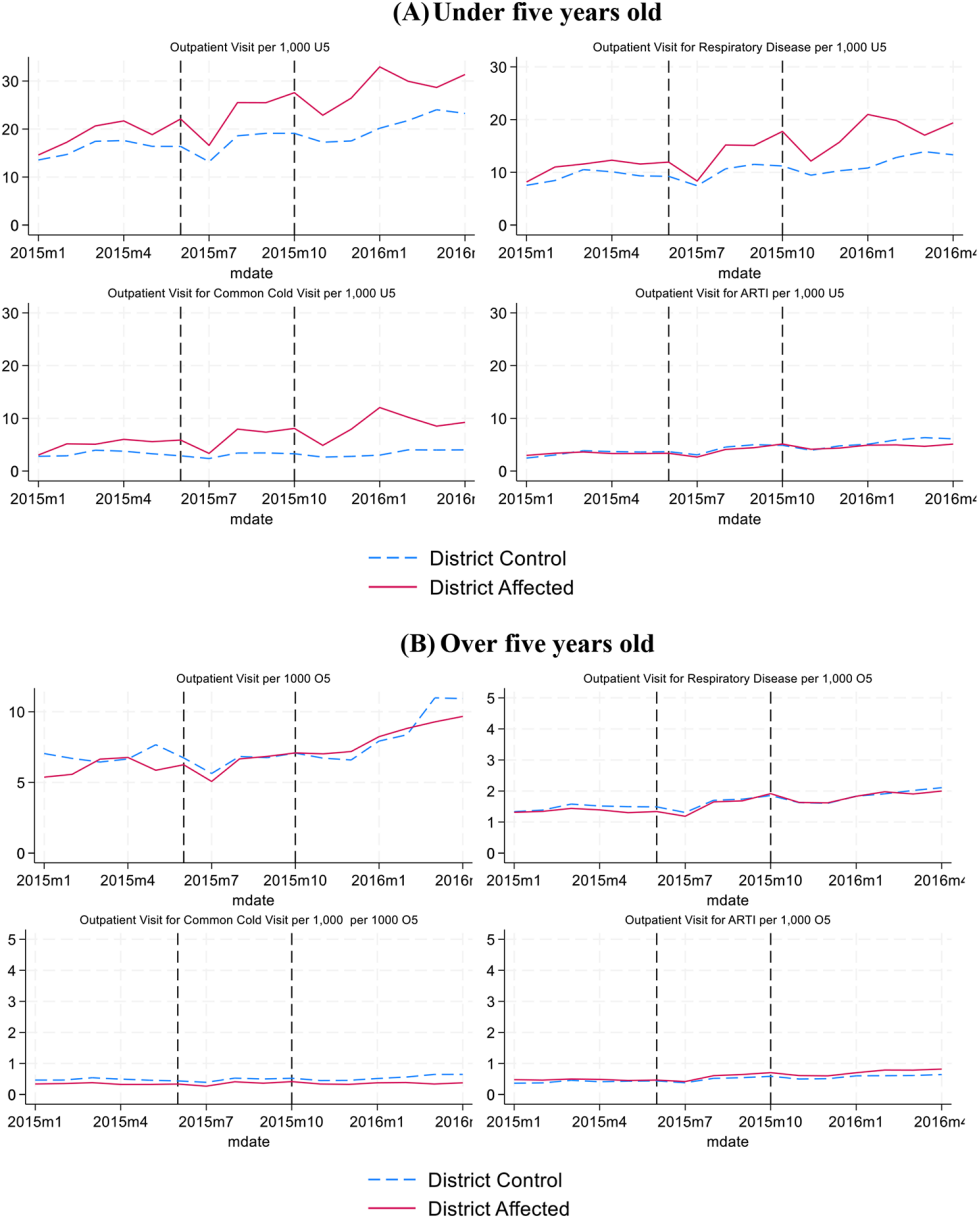
Primary care outpatient and respiratory disease trend in Sumatera and Kalimantan Islands (Monthly visit per 1,000 age group enrollees, 2015–2016). *Source*: Author analysis based on BPJS Kesehatan data. *Note*: District affected is a district with average monthly AOD value more than 0.75 during Forest Fire Period (June to October 2015)

**Fig. 4:**
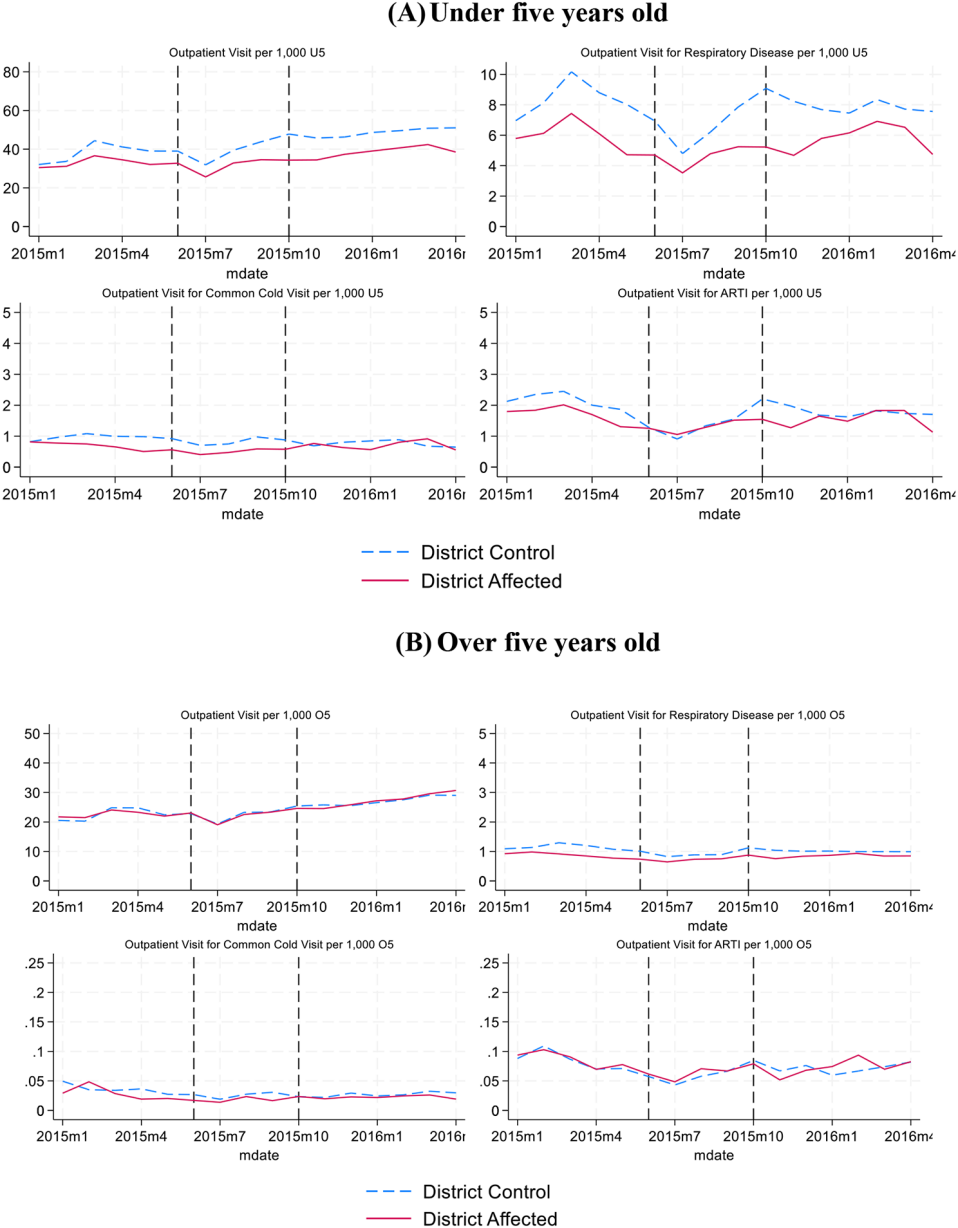
Hospital care outpatient and respiratory disease trend in Sumatera and Kalimantan Islands (Monthly visit per 1,000 age group enrollees, 2015–2016). *Source*: Author analysis based on BPJS Kesehatan data. *Note*: District affected is a district with average monthly AOD value more than 0.75 during Forest Fire Period (June to October 2015)

Figures 3 and 4 show trends of primary care and hospital care use in affected and control districts, for (A) under five years old and (B) over five years old. In Fig. 3A, we see an increase during and after the forest fires for total visits, respiratory disease and common cold visits in a primary care facility for under five years old in affected districts. Control districts also experience an increase in outpatient care, but not as strong as in affected districts. For those age five years and older, we observe some increase in utilization but no difference in trends between affected and control districts. Figure 4A and B show that affected districts have relatively lower hospital outpatient visit rates, but the trends are fairly similar to those for the control districts, with a dip in visits for respiratory disease during the forest fire period.

To further assess the parallel trends assumption, we compare the monthly changes in the outcome variables in the pre-fire period, by interacting month dummies variables with the variable indicating affected status. We find that none of the coefficients for the interactions are statistically significant (Appendix 11 A and 11B). We also perform a joint significance test of the interaction terms of time trend and forest fire affected status using the joint F-test, which never rejects the null hypothesis that the pre-fire time trends are significantly different in to be treated and controls (Appendix 12). This leads us to conclude that while there are some initial differences in outcomes between the affected and control group, the trends for these groups are similar before the forest fires occurred. We believe that this builds some confidence in the parallel trends assumption for the period after June 2015.

Our main ANCOVA estimation results are presented in Table 2 for outpatient care in primary care facilities and in Table 3 for hospitals. The forest fires are estimated to have caused an immediate increased utilization of primary care in affected districts by 1.42 visits (but not statistically significant), and a delayed increase of 3.13 visits after the fire period, per 1,000 under five enrollees for total primary care visit. This means a 17% increase of total primary visits after forest fire compared to pre-fire utilization levels.^2^ When we look at the causes for the outpatient visits, we find statistically significant effects after the forest fire period for respiratory disease (2,41 visits per 1,000) and common cold (2.14), which translates to increases of respectively 22% and 43%.

However, we find opposite results for utilization at hospital for under-fives, with visit rates decreasing by 3.26 during the fires and 5.15 afterwards (Table 3), which constitutes a 10% and 16% drop relative to observed pre-fire utilization levels in the affected districts. For the population over five years old there appears to have been no discernable impacts of forest fire on health care use.

Our geographic breakdown shows that the impact of the fires on primary healthcare use is mainly due to utilization changes in urban rather than in rural areas. For example, we find consistent positive effects of forest fire on overall utilization, respiratory disease and common cold for under-fives (Table 4). However, for hospital care we observe negative effects for both urban and rural districts, although the estimates for urban districts are less precise (Table 5).

**Table 1:** Summary statistics and district baseline characteristics

	Pre-Fire			During Fire			Post-Fire		
	Affected (A)	Control (C)	Diff (A-C)	Affected (A)	Control (C)	Diff (A-C)	Affected (A)	Control (C)	Diff (A-C)
Variable	Mean	Mean		Mean	Mean		Mean	Mean	
**Monthly Healthcare Use in Primary Care**									
All Age									
Total outpatient visits per 1,000 enrolees	6.26	7.13	-0.87	6.65	6.88	-0.23	8.65	8.89	-0.25
Respiratory disease visits per 1,000 enrolees	1.56	1.66	-0.11	1.78	1.84	-0.06	2.07	2.11	-0.04
Common cold visit per 1,000 enrolees	0.40	0.56	-0.15**	0.42	0.54	-0.12*	0.42	0.61	-0.20***
ARTI visit per 1,000 enrolees	0.55	0.49	0.07	0.66	0.59	0.07	0.82	0.70	0.12
Under 5									
Total outpatient visits per 1,000 under 5 enrolees	18.60	15.92	2.68	23.45	17.27	6.18	28.69	20.68	8.02
Respiratory disease visits per 1,000 under 5 enrolees	10.92	9.18	1.74	13.66	10.01	3.65	17.50	11.78	5.72
Common cold visit per 1,000 under 5 enrolees	4.98	3.33	1.64	6.53	3.08	3.44	8.79	3.42	5.38
ARTI visit per 1,000 under 5 enrolees	0.55	0.49	0.06	0.66	0.59	0.07	0.82	0.70	0.12
**Monthly Healthcare Use in Hospital Care**									
All Age									
Total outpatient visits per 1,000 enrolees	22.73	22.90	-0.16	22.71	23.25	-0.54	27.82	27.77	0.05
Respiratory disease outpatient visits per 1,000 enrolees	1.03	1.35	-0.32**	0.86	1.10	-0.24**	0.98	1.18	-0.20*
Common cold outpatient visits per 1,000 enrolees	0.05	0.06	-0.01	0.03	0.05	-0.01*	0.04	0.05	-0.01
ARTI outpatient visits per 1,000 enrolees	0.13	0.14	-0.01	0.10	0.10	0.00	0.11	0.11	-0.00
Under 5									
Total outpatient visits per 1,000 under 5 enrolees	32.96	38.02	-5.06*	32.03	40.40	-8.37***	38.75	48.71	-9.96***
Respiratory disease outpatient visits per 1,000 under 5 enrolees	6.03	8.40	-2.37***	4.70	6.98	-2.28***	5.80	7.83	-2.03***
Common cold outpatient visits per 1,000 under 5 enrolees	0.70	0.97	-0.27*	0.52	0.85	-0.32**	0.71	0.76	-0.05
ARTI outpatient visits per 1,000 under 5 enrolees	1.73	2.16	-0.43*	1.33	1.45	-0.12	1.53	1.76	-0.22
Aerosol Optical Depth (AOD)									
Mean Value	0.30	0.28	0.02***	1.12	0.52	0.60***	0.33	0.32	0.01**
District Baseline Character									
Doctors per 1,000 JKN member (January 2015)	0.29	0.35	-0.055*						
Average district JKN member (January 2015)	169,620	179,069	-9,449						

Source: Healthcare use district aggregated from BPJS Kesehatan data. Aerosol Optical Depth derived from MODIS satellite data. District Baseline Characteristics comes from Susenas 2015, PODES 2014 and Indonesian Statistics Office (BPS). Note: District affected is a district with 5-month average AOD value more than 0.75 during forest fire period. Pre-Fire (January-May 2015); During Fire (June-October 2015); After Fire (November 2015 – April 2016)

**Table 2:** The effect of forest fire affected districts on primary care utilization 2015–2016 - ANCOVA

	Under Five Years Old				Over Five Years Old			
	(1)	(2)	(3)	(4)	(5)	(6)	(7)	(8)
	Total Visit per 1,000 Under Five members	Respiratory visits per 1,000 Under Five members	Common Cold visit per 1,000 Under Five members	ARTI visit per 1,000 Under Five members	Total Visit per 1,000 Over Five members	Respiratory visits per 1,000 Over Five members	Common Cold visit per 1,000 Over Five members	ARTI visit per 1,000 Over Five members
During Forest Fire	1.421	0.432	0.264	-0.277	0.160	0.0312	-0.0135	0.00239
	(1.408)	(1.089)	(0.791)	(0.269)	(0.259)	(0.0606)	(0.0258)	(0.0295)
After Forest Fire	3.130^*^	2.406^*^	2.136^***^	-0.655	0.312	0.0679	-0.0845^***^	0.0661
	(1.634)	(1.251)	(0.801)	(0.406)	(0.347)	(0.0822)	(0.0290)	(0.0447)
Pre-Fire Outcome (January-May 2015)	1.743^***^	1.847^***^	1.920^***^	1.120^***^	1.032^***^	0.957^***^	0.751^***^	1.053^***^
	(0.137)	(0.217)	(0.234)	(0.0328)	(0.0703)	(0.0249)	(0.0254)	(0.0325)
Constant	-11.74^***^	-8.349^***^	-3.808^***^	-0.0807	-0.477	0.0336	0.0828^***^	-0.0193
	(2.765)	(2.404)	(1.353)	(0.276)	(0.483)	(0.0711)	(0.0310)	(0.0307)
Time Dummies	Y	Y	Y	Y	Y	Y	Y	Y
N: District-Month	2,203	2,203	2,203	2,203	2,203	2,203	2,203	2,203

Standard errors in parentheses * *p* < 0.10; ** *p* < 0.05; *** *p* < 0.01

Note: District JKN Member use January 2015 JKN member per district figure as a baseline

**Table 3:** The effect of forest fire affected districts on hospital utilization 2015–2016 - ANCOVA

	Under Five Years Old				Over Five Years Old			
	(1)	(2)	(3)	(4)	(5)	(6)	(7)	(8)
	Total Visit per 1,000 Under Five members	Respiratory visits per 1,000 Under Five members	Common Cold visit per 1,000 Under Five members	ARTI visit per 1,000 Under Five members	Total Visit per 1,000 Over Five members	Respiratory visits per 1,000 Over Five members	Common Cold visit per 1,000 Over Five members	ARTI visit per 1,000 Over Five members
During Forest Fire	-3.261^***^	-0.275	-0.132	0.170	-0.200	0.00542	-0.00289	0.00213
	(0.951)	(0.318)	(0.0812)	(0.105)	(0.396)	(0.0343)	(0.00301)	(0.00555)
After Forest Fire	-5.147^***^	-0.0767	0.141^*^	0.0606	0.235	0.0377	-0.00145	0.000518
	(1.244)	(0.298)	(0.0791)	(0.127)	(0.454)	(0.0394)	(0.00257)	(0.00630)
Pre-Fire Outcome (January-May 2015)	0.979^***^	0.829^***^	0.704^***^	0.670^***^	1.032^***^	0.737^***^	0.495^***^	0.853^***^
	(0.0149)	(0.0306)	(0.0423)	(0.0383)	(0.0125)	(0.0253)	(0.0579)	(0.108)
Constant	2.831^***^	-0.0410	0.215^**^	-0.113	-0.222	0.108^***^	0.00675^*^	-0.0152
	(1.030)	(0.333)	(0.0915)	(0.117)	(0.466)	(0.0412)	(0.00350)	(0.0114)
Time Dummies	Y	Y	Y	Y	Y	Y	Y	Y
N: District-Month	2,203	2,203	2,203	2,203	2,203	2,203	2,203	2,203

Standard errors in parentheses * *p* < 0.10; ** *p* < 0.05; *** *p* < 0.01

Note: District JKN Member use January 2015 JKN member per district figure as a baseline

**Table 4:** The effect of forest fire affected districts on primary care utilization 2015–2016 under five years old (urban v– rural) - ANCOVA

	City + Regency				City (urban)				Regency (rural)			
	(1)	(2)	(3)	(4)	(5)	(6)	(7)	(8)	(9)	(10)	(11)	(12)
	Total Visit per 1,000 Under Five Years JKN members	Respiratory visits per 1,000 Under Five Years JKN members	Common Cold visit per 1,000 Under Five JKN Years members	ARTI visit per 1,000 Under Five Years JKN members	Total Visit per 1,000 Under Five Years JKN members	Respiratory visits per 1,000 Under Five Years JKN members	Common Cold visit per 1,000 Under Five JKN Years members	ARTI visit per 1,000 Under Five Years JKN members	Total Visit per 1,000 Under Five Years JKN members	Respiratory visits per 1,000 Under Five Years JKN members	Common Cold visit per 1,000 Under Five JKN Years members	ARTI visit per 1,000 Under Five Years JKN members
During Forest Fire	1.421	0.432	0.264	-0.277	5.798^***^	3.283^***^	1.405^***^	0.252	0.128	-0.619	-0.648	-0.345
	(1.408)	(1.089)	(0.791)	(0.269)	(1.410)	(1.026)	(0.448)	(0.651)	(1.550)	(1.198)	(0.946)	(0.287)
After Forest Fire	3.130^*^	2.406^*^	2.136^***^	-0.655	5.924^***^	2.910^**^	0.748	-0.242	2.347	2.035	1.926^**^	-0.695
	(1.634)	(1.251)	(0.801)	(0.406)	(1.939)	(1.326)	(0.532)	(0.796)	(1.697)	(1.258)	(0.892)	(0.466)
Pre-Fire Outcome (January-May 2015)	1.743^***^	1.847^***^	1.920^***^	1.120^***^	0.997^***^	0.938^***^	0.610^***^	1.114^***^	1.855^***^	1.995^***^	1.941^***^	1.193^***^
	(0.137)	(0.217)	(0.234)	(0.0328)	(0.0328)	(0.0407)	(0.0526)	(0.0447)	(0.147)	(0.242)	(0.237)	(0.0475)
Time Dummies	Y	Y	Y	Y	Y	Y	Y	Y	Y	Y	Y	Y
N: District-Month	2,203	2,203	2,203	2,203	451	451	451	451	1,752	1,752	1,752	1,752

Standard errors in parentheses * *p* < 0.10; ** *p* < 0.05; *** *p* < 0.01

Note: District JKN Member use January 2015 JKN member per district figure as a baseline

**Table 5:** The Effect of forest fire affected districts on hospital care utilization under five years old (urban v– rural) - ANCOVA

	City + Regency				City (urban)				Regency (rural)			
	(1)	(2)	(3)	(4)	(5)	(6)	(7)	(8)	(9)	(10)	(11)	(12)
	Total Visit per 1,000 Under Five Years JKN members	Respiratory visits per 1,000 Under Five Years JKN members	Common Cold visit per 1,000 Under Five JKN Years members	ARTI visit per 1,000 Under Five Years JKN members	Total Visit per 1,000 Under Five Years JKN members	Respiratory visits per 1,000 Under Five Years JKN members	Common Cold visit per 1,000 Under Five JKN Years members	ARTI visit per 1,000 Under Five Years JKN members	Total Visit per 1,000 Under Five Years JKN members	Respiratory visits per 1,000 Under Five Years JKN members	Common Cold visit per 1,000 Under Five JKN Years members	ARTI visit per 1,000 Under Five Years JKN members
During Forest Fire	-3.261^***^	-0.275	-0.132	0.170	-2.870	-0.235	0.131	0.0956	-3.493^***^	-0.338	-0.198^**^	0.182
	(0.951)	(0.318)	(0.0812)	(0.105)	(2.806)	(0.605)	(0.157)	(0.181)	(0.956)	(0.365)	(0.0927)	(0.122)
After Forest Fire	-5.147^***^	-0.0767	0.141^*^	0.0606	-6.362	-2.415^***^	-0.152	-0.732^***^	-5.415^***^	0.407	0.207^**^	0.238
	(1.244)	(0.298)	(0.0791)	(0.127)	(4.457)	(0.682)	(0.150)	(0.260)	(1.103)	(0.330)	(0.0918)	(0.145)
Pre-Fire Outcome (January-May 2015)	0.979^***^	0.829^***^	0.704^***^	0.670^***^	0.914^***^	0.681^***^	0.649^***^	0.692^***^	0.959^***^	0.847^***^	0.708^***^	0.663^***^
	(0.0149)	(0.0306)	(0.0423)	(0.0383)	(0.0231)	(0.0388)	(0.0896)	(0.0493)	(0.0195)	(0.0353)	(0.0438)	(0.0444)
Time Dummies	Y	Y	Y	Y	Y	Y	Y	Y	Y	Y	Y	Y
N: District-Month	2,203	2,203	2,203	2,203	451	451	451	451	1,752	1,752	1,752	1,752

## Discussion

Our analysis has led to at least five clear findings, all related to the four important distinctions that we make: respiratory versus other health care use, young (< 5y) versus older (> 5y) population, urban versus rural regions, and effects during versus after the forest fires.

*First*, perhaps the most important observation is that for the large majority of the 5 + population, *no effects* on respiratory or other health care utilization could be detected of the forest fires, neither in the short (5 months) period during, nor in the (6 months) period right after the forest fires: the fires do not seem to have caused the run on respiratory and/or other health care that one might expect given the reported longer term health outcomes. We find no short-term effects on either primary (clinic) or secondary (hospital) health care utilization of > 5y olds, neither during nor after the fires. That may be surprising in view of the documented longer-term effects on health and raises questions about the ability of adequate (short term) health care use in preventing longer term health consequences on morbidity and mortality observed in Indonesia and elsewhere, but most of these reported effects were also for children [5, 30].

*Secondly*, we do find some positive health care utilization effects for under 5y olds, but they are opposite for primary care (which increases) and secondary care, (which decreases). The latter decrease is only observed for total hospital visits, not for respiratory disease specific visits. It raises questions about whether the fires may have had any indirect health impact through foregone other care use for children, i.e. for other than respiratory problems, as a result of reduced accessibility. Our data do not allow us to examine this possibility other than through a separate estimation for urban and rural parts of the country.

We use regency as a proxy of rural area and city for urban area. According to Kompas [31] regencies typically encompass a larger area compared to cities, resulting in a higher number of disadvantaged villages within district boundaries. Moreover, regencies tend to have lower population densities compared to cities. In terms of livelihood, regency residents commonly engage in agriculture, while those in city are more involved in trade and services. In socio-cultural aspect, city residents often exhibit higher levels of education and better health outcomes compared to their counterparts in districts. Additionally, public service facilities (including education and health) are typically more developed in cities than in regencies. In the economic point of view, the average Gross Regional Domestic Product (GRDP) is lower in regencies than in cities.

This led to a *third* finding: the increase in primary care for young children is almost exclusively observed in the urban areas, not in the rural. It is only in urban clinics that we observe substantial increases both during and after the forest fires. This again points in the direction of only rural kids’ primary care use not responding to the fire smoke exposure. Whether this is a consequence of a deliberate rural mother/parent choice not to seek care during/after the fires, or due to a more general disruption of primary care services in affected rural areas is something we cannot derive from our data.

The same urban-rural breakdown, but for children’s hospital care use, led to a *fourth* finding: it revealed that almost the entire observed drop in total (i.e. non respiratory specific) visits is due to a reduction in *rural* hospital visits. Again, we argue that accessibility of hospitals during fires is much more restricted for rural than for urban mothers wanting to take their under 5s to the hospital for reasons unrelated to respiratory problems. If this is indeed the case, then it suggests that the health (care) burden deriving from smoke pollution is very unequally distributed, and far greater in rural than in urban areas.

Fifth and finally, the only instance where we see a clear difference in effects during versus after the fires is in common cold visits for children: again the significant rise in common cold visits *after* the fires is almost entirely due to a rise in rural clinic visits. This may be partly due to a catch-up effect for the significant drop that was observed *during* the fires. While it is surprising to see this for common cold visits and not for the more serious category of ARTI visits, we have to be aware that some of the visits labeled as common cold might simply be misclassifications or might sometimes develop into more serious conditions like ARTI later.

Our results contribute to the rather small literature on the health care utilization impacts of wildfire smoke, which has mostly focused on developed countries. Our results contrast to a similar study of Ye et al. (2021) which investigates hospital admissions resulting from wildfire smoke but for a much longer period (2000–2015) in Brazil. They find increased utilization of hospital services while our analysis indicates a decrease. A plausible explanation is that our study focuses on the much shorter intense period of forest fires which is likely to also have limited access through reduced mobility while the Brazil study has a much longer time frame. Ye et al. (2021) also find the strongest effects among children. Our findings also differ from those of Sheldon and Sankaran (2017) for Singapore, who found that an increase in pollution led to an increase in the frequency of weekly polyclinic visits for ARTIs while we do not find any increase in ARTI visits.

Our study is subject to various limitations. First, the AOD data per district are only a proxy measure for the PM2.5 concentrations which have been used in other health studies. Problems like cloud contamination, varied surface conditions, or flawed retrievals may result in inaccuracies or absence of the AOD values retrieved from the satellite (Handschuh et al., 2022). This may lead to a downward bias in our effect estimates. Second, our main outcome variable – health care visits – only relate to the JKN members in the two islands (Kalimantan and Sumatra). This is only 57% of the total Sumatera and Kalimantan resident population. Finally, the reasons indicated for the visits in the insurance administration (common cold, ARTI, other) may be subject to classification errors that could influence our findings.

## Conclusion

This study aimed to estimate the short-term consequences of the forest fires that (two islands of) Indonesia endured from June to October 2015, for the use of health care at primary facilities and hospitals. We find that the health care utilization of the 5 + population was not affected but in the more urban places, parents did take their children to the primary care clinics in response to fire smoke and the observed drop in (both general and respiratory specific) hospital care use is generally not significant. We interpret this finding of forgone care as suggesting that the accessibility of care, whether perceived or real, was primarily restricted to rural areas, while it was less observed, if at all, during the fires in urban areas. It is quite possible that seeking care during times of fire and smoke is more hindered in rural than in urban communities. Foregone care is a situation where individuals either opt not to or are unable to access health services, even when they recognize a need for them [32]. This raises equity issues as it would suggest that the effects of foregone care use are unequally distributed between urban and rural Indonesia. Our findings underline the importance of having available medical services in proximity during a period of forest fires, when transport and mobility is hindered by the same fires.

The reduced use of general hospital care for kids does, however, also raise important questions about the potential harmful effects of foregone care for other than respiratory reasons. Our analysis does not allow us to examine these longer-term health consequences, but if the foregone care use was essential, this is a possibility. The most important implication for health policy appears to be that in post-fire periods additional attention is needed for catching up with essential care for kids, especially in rural areas.

Indonesia has made commendable efforts to document and manage these natural disasters through agencies, such as the National Disaster Management Agency (BNPB) and the Ministry of Forestry and Environment. These organizations collect comprehensive data on the impact of disasters, encompassing affected regions, population demographics, and environmental conditions. However, this effort is fragmented and requires synchronisation in order to make these data more valuable for research. Thus, our present analysis is limited to the data that were available.

We recommend future synchronisation of healthcare data from BPJS Kesehatan and natural hazard data. As a result, researchers and policymakers could gain valuable insights into several critical areas. For instance, they could explore the effectiveness of healthcare responses during and after disasters, identify vulnerable populations, assess healthcare infrastructure readiness, and develop strategies to enhance healthcare preparedness in disaster-prone regions.

### Electronic supplementary material

Below is the link to the electronic supplementary material.

Supplementary Material 1

## Data Availability

The research relies on anonymized secondary administrative data obtained from BPJS Kesehatan, accessible through formal requests made to the agency.

## Footnotes

1 GEE is geospatial analysis software that collect and examine satellite photos of our world. We can access this database through the link https://earthengine.google.com/. Scientists and non-profits use Earth Engine for remote sensing research, predicting disease outbreaks, natural resource management, and more. We use user-friendly data environment through https://code.earthengine.google.com/.

2 The DID estimates in Appendix 3 show similar results, albeit larger point estimates.

## Abbreviations

AOD: Aerosol Optical Depth
ARTI: Acute Respiratory Tract Infection
ANCOVA: Analysis of Covariance
CO: Carbon monoxide
CO_2_: Carbon dioxide
COPD: Chronic obstructive pulmonary disease, or COPD)
GADM: Global Administrative Areas
GEE: Google Earth Engine system
ICD10: International Classification of Diseases 10th Revision
MODIS: Moderate-Resolution Imaging Spectroradiometer
Nox: Nitrogen oxides
PAH: Polycyclic aromatic hydrocarbon (PAH)
PM 2.5: Particulate matter size 0.1 to 2.5 micrometers
PM 210: Particulate matter size 2.5 to 10 micrometers
SO2: Sulphur dioxide
