# Supplementary material for: When the smoke gets in your lungs: short-term effects of Indonesia’s 2015 forest fires on health care use
Source: Environ Health. 2024 May 3;23:44. doi: 10.1186/s12940-024-01079-x (PMC11067070; doi:10.1186/s12940-024-01079-x)
Supplement: Supplementary file 1 — Supplementary Material 1 [file 12940_2024_1079_MOESM1_ESM.docx]

### Appendix 1. Geospatial Data Interpolation

We find that our AOD data downloaded from GEE has a large number of missing values (55 percent). This could affect our estimation if these missing are nonrandom. We use nearby districts’ AOD values to predict the missing values. The spatial interpolation process that we use creates a imputed value from nearby sample points by creating a surface based on values at isolated sample points (1). Geospatial interpolation can be used to fill missing values of any spatially based information such as rainfall, fire, dust and pollution with the predicted value. We choose local neighbourhood approach interpolation called Inverse Distance Weighting (IDW). IDW is predicated on the idea that a value at an unsampled point can be roughly estimated as a weighted average of values at points within a specific cut-off distance or from a set number of $s$ of the points (2). We only interpolate across space and not do this approach across time. We use the following specification to calculate a missing AOD observation in certain site using this estimation:

$\hat{{AOD}_{st}} = \frac{\sum_{i} \omega_{i}{yAOD}_{it}}{\sum_{i} \omega_{i}}$ (1)

Where the missing AOD at district $s$in month $t$ is replaced by a weighted average of the observed AOD values of its neighboring districts denoted with the subscript $i$ at the same month $t$. $\hat{{AOD}_{st}}$ is the imputed value AOD at district $s$ at month $t$ . $\omega_{i}$is equal to $\frac{1}{d_{i}{(s)}^{2}}$ and $d_{i}{(s)}^{2}$ is the squared of the Euclidean distance between district $i$and district $s$ (3). We include all districts which are within a 15 km threshold of districts with missing data. The approach provides the highest weights to the closest neighbors in determining the imputed AOD level for the missing district $s$. To perform IDW interpolation, we use QGIS toolbox available in the software.

To assess the accuracy of our interpolated AOD values, we compared them with some part of observed values to get the residuals. Our approach involved splitting the raw data (neighbouring areas) into 80% for prediction and 20% for validation. We then conducted interpolation using 80% of the data and validated it with the remaining 20%. The difference between these two are the residuals. From these residuals we can calculate error metrics such as root mean squared error (RMSE). A low RMSE indicates that the IDW interpolations are close to the actual values, indicating a better fit of the model to the data and more precise. Conversely, higher RMSE values suggest that the model's predictions are farther from the actual values, indicating more error and less precision in the predictions. We found that our RMSE is 0.133 meaning that our IDW interpolation data is close to the observed value in the validation sample.

### Appendix 2. Average AOD Trend in Sumatera and Kalimantan Islands (District Affected vs District Control)

Cut off AOD=0.75

Cut off AOD=0.50

Source: Author analysis based on AOD value from MODIS Data.

**Note**: District affected is a district with an AOD 5-month average value > 0.75 during forest fire period; District control is a district with an AOD 5-month average value < 0.75 during forest fire period (June to October 2015). We use a threshold of AOD equal to 0.50 for a robustness check and comparison. We find that even with the lower threshold, there is still a small rise in critical AOD visible in the control districts, but less so than with the higher (0.75) threshold. This highlights the fact that any arbitrary threshold will show some spillover effect of the smoke of forest fires in neighbouring districts. The difference between treated and controls, however, remains similar.

### Appendix 3. The Effect of Forest Fire Affected Districts on Primary Care Utilization 2015-2016 (Standard DID)

|  | Under Five Years Old | | | | Over Five Years Old | | | |
| --- | --- | --- | --- | --- | --- | --- | --- | --- |
|  | (1) | (2) | (3) | (4) | (5) | (6) | (7) | (8) |
|  | Total Visit per 1,000 Under Five members | Respiratory visits per 1,000 Under Five members | Common Cold visit per 1,000 Under Five members | ARTI visit per 1,000 Under Five members | Total Visit per 1,000 Over Five members | Respiratory visits per 1,000 Over Five members | Common Cold visit per 1,000 Over Five members | ARTI visit per 1,000 Over Five members |
| During Forest Fire | 3.442^*^ | 1.888 | 1.775 | -0.281 | 0.0793 | 0.0274 | 0.0156 | 0.00586 |
|  | (1.821) | (1.402) | (1.204) | (0.331) | (0.370) | (0.0717) | (0.0315) | (0.0370) |
| After Forest Fire | 5.164^**^ | 3.889^**^ | 3.667^**^ | -0.664^*^ | 0.241 | 0.0644 | -0.0537 | 0.0682^*^ |
|  | (2.213) | (1.744) | (1.518) | (0.370) | (0.365) | (0.0768) | (0.0329) | (0.0395) |
| Time Dummies | Y | Y | Y | Y | Y | Y | Y | Y |
| District Fixed Effect | Y | Y | Y | Y | Y | Y | Y | Y |
| N: District-Month | 3,216 | 3,216 | 3,216 | 3,216 | 3,216 | 3,216 | 3,216 | 3,216 |

### Appendix 4. The Effect of Forest Fire Affected Districts on Hospital Care Utilization 2015-2016 (Standard DID)

|  | Under Five Years Old | | | | Over Five Years Old | | | |
| --- | --- | --- | --- | --- | --- | --- | --- | --- |
|  | (1) | (2) | (3) | (4) | (5) | (6) | (7) | (8) |
|  | Total Visit per 1,000 Under Five members | Respiratory visits per 1,000 Under Five members | Common Cold visit per 1,000 Under Five members | ARTI visit per 1,000 Under Five members | Total Visit per 1,000 Over Five members | Respiratory visits per 1,000 Over Five members | Common Cold visit per 1,000 Over Five members | ARTI visit per 1,000 Over Five members |
| During Forest Fire | -3.145^***^ | 0.138 | -0.0511 | 0.314^**^ | -0.204 | 0.0777 | 0.000928 | 0.00187 |
|  | (1.169) | (0.410) | (0.110) | (0.151) | (0.465) | (0.0478) | (0.00666) | (0.00713) |
| After Forest Fire | -4.990^***^ | 0.344 | 0.220^**^ | 0.203 | 0.258 | 0.110^**^ | 0.00232 | 0.000321 |
|  | (1.255) | (0.392) | (0.109) | (0.160) | (0.479) | (0.0461) | (0.00641) | (0.00752) |
| Time Dummies | Y | Y | Y | Y | Y | Y | Y | Y |
| District Fixed Effect | Y | Y | Y | Y | Y | Y | Y | Y |
| N: District-Month | 3,216 | 3,216 | 3,216 | 3,216 | 3,216 | 3,216 | 3,216 | 3,216 |

Standard errors in parentheses * p<0.10; ** p<0.05; *** p<0.01

Note: District JKN Member use January 2015 JKN member per district figure as a baseline.

### Appendix 5. The Effect of forest fire affected districts on primary care utilization under five years old (urban vs rural) (Standard DID)

|  | City + Regency | | | | City (urban) | | | | Regency (rural) | | | |
| --- | --- | --- | --- | --- | --- | --- | --- | --- | --- | --- | --- | --- |
|  | (1) | (2) | (3) | (4) | (5) | (6) | (7) | (8) | (9) | (10) | (11) | (12) |
|  | Total Visit per 1,000 Under Five Years JKN members | Respiratory visits per 1,000 Under Five Years JKN members | Common Cold visit per 1,000 Under Five JKN Years members | ARTI visit per 1,000 Under Five Years JKN members | Total Visit per 1,000 Under Five Years JKN members | Respiratory visits per 1,000 Under Five Years JKN members | Common Cold visit per 1,000 Under Five JKN Years members | ARTI visit per 1,000 Under Five Years JKN members | Total Visit per 1,000 Under Five Years JKN members | Respiratory visits per 1,000 Under Five Years JKN members | Common Cold visit per 1,000 Under Five JKN Years members | ARTI visit per 1,000 Under Five Years JKN members |
| During Forest Fire | 3.442^*^ | 1.888 | 1.775 | -0.281 | 5.804^***^ | 3.401^**^ | 2.247^***^ | 0.229 | 3.030 | 1.645 | 1.734 | -0.384 |
|  | (1.821) | (1.402) | (1.204) | (0.331) | (2.020) | (1.387) | (0.657) | (0.842) | (2.256) | (1.749) | (1.526) | (0.357) |
| After Forest Fire | 5.164^**^ | 3.889^**^ | 3.667^**^ | -0.664^*^ | 5.930^***^ | 3.028^**^ | 1.590^**^ | -0.266 | 5.259^*^ | 4.333^**^ | 4.336^**^ | -0.742^*^ |
|  | (2.213) | (1.744) | (1.518) | (0.370) | (2.176) | (1.505) | (0.715) | (0.927) | (2.765) | (2.191) | (1.931) | (0.404) |
| District Fixed Effect | Y | Y | Y | Y | Y | Y | Y | Y | Y | Y | Y | Y |
| Time Dummies | Y | Y | Y | Y | Y | Y | Y | Y | Y | Y | Y | Y |
| N: District-Month | 3,216 | 3,216 | 3,216 | 3,216 | 656 | 656 | 656 | 656 | 2,560 | 2,560 | 2,560 | 2,560 |

Standard errors in parentheses * p<0.10; ** p<0.05; *** p<0.01

Note: District JKN Member use January 2015 JKN member per district figure as a baseline.

### Appendix 6. The Effect of forest fire affected districts on hospital care utilization under five years old (urban vs rural) (Standard DID)

|  | City + Regency | | | | City (urban) | | | | Regency (rural) | | | |
| --- | --- | --- | --- | --- | --- | --- | --- | --- | --- | --- | --- | --- |
|  | (1) | (2) | (3) | (4) | (5) | (6) | (7) | (8) | (9) | (10) | (11) | (12) |
|  | Total Visit per 1,000 Under Five Years enrolees | Respiratory visits per 1,000 Under Five Years enrolees | Common Cold visit per 1,000 Under Five Years enrolees | ARTI visit per 1,000 Under Five Years enrolees | Total Visit per 1,000 Under Five Years enrolees | Respiratory visits per 1,000 Under Five Years enrolees | Common Cold visit per 1,000 Under Five Years enrolees | ARTI visit per 1,000 Under Five Years enrolees | Total Visit per 1,000 Under Five Years enrolees | Respiratory visits per 1,000 Under Five Years enrolees | Common Cold visit per 1,000 Under Five Years enrolees | ARTI visit per 1,000 Under Five Years enrolees |
| During Forest Fire | -3.145^***^ | 0.138 | -0.0511 | 0.314^**^ | -3.489 | 0.595 | 0.164 | 0.176 | -3.090^***^ | 0.0371 | -0.109 | 0.352^**^ |
|  | (1.169) | (0.410) | (0.110) | (0.151) | (3.419) | (0.760) | (0.183) | (0.286) | (1.188) | (0.473) | (0.129) | (0.174) |
| After Forest Fire | -4.990^***^ | 0.344 | 0.220^**^ | 0.203 | -6.981^*^ | -1.586^**^ | -0.119 | -0.651^**^ | -4.962^***^ | 0.793^*^ | 0.294^**^ | 0.407^**^ |
|  | (1.255) | (0.392) | (0.109) | (0.160) | (3.940) | (0.746) | (0.174) | (0.320) | (1.225) | (0.451) | (0.128) | (0.183) |
| District Fixed Effect | Y | Y | Y | Y | Y | Y | Y | Y | Y | Y | Y | Y |
| Time Dummies | Y | Y | Y | Y | Y | Y | Y | Y | Y | Y | Y | Y |
| N: District-Month | 3,216 | 3,216 | 3,216 | 3,216 | 656 | 656 | 656 | 656 | 2,560 | 2,560 | 2,560 | 2,560 |

Standard errors in parentheses * p<0.10; ** p<0.05; *** p<0.01

Note: District JKN Member use January 2015 JKN member per district figure as a baseline.

### Appendix 7. The Effect of forest fire affected districts on primary care utilization over five years old (urban vs rural) (ANCOVA Regression)

|  | City + Regency | | | | City (urban) | | | | Regency (rural) | | | |
| --- | --- | --- | --- | --- | --- | --- | --- | --- | --- | --- | --- | --- |
|  | (1) | (2) | (3) | (4) | (5) | (6) | (7) | (8) | (9) | (10) | (11) | (12) |
|  | Total Visit per 1,000 Under Five Years JKN members | Respiratory visits per 1,000 Under Five Years JKN members | Common Cold visit per 1,000 Under Five JKN Years members | ARTI visit per 1,000 Under Five Years JKN members | Total Visit per 1,000 Under Five Years JKN members | Respiratory visits per 1,000 Under Five Years JKN members | Common Cold visit per 1,000 Under Five JKN Years members | ARTI visit per 1,000 Under Five Years JKN members | Total Visit per 1,000 Under Five Years JKN members | Respiratory visits per 1,000 Under Five Years JKN members | Common Cold visit per 1,000 Under Five JKN Years members | ARTI visit per 1,000 Under Five Years JKN members |
| During Forest Fire | 0.160 | 0.0312 | -0.0135 | 0.00239 | 1.756^***^ | 0.690^***^ | 0.0927 | 0.197^***^ | -0.0168 | -0.0709 | -0.0298 | -0.0220 |
|  | (0.259) | (0.0606) | (0.0258) | (0.0295) | (0.481) | (0.170) | (0.0665) | (0.0757) | (0.298) | (0.0603) | (0.0266) | (0.0295) |
| After Forest Fire | 0.312 | 0.0679 | -0.0845^***^ | 0.0661 | 4.046^***^ | 1.036^***^ | 0.0469 | 0.284^***^ | -0.351 | -0.104 | -0.106^***^ | 0.0413 |
|  | (0.347) | (0.0822) | (0.0290) | (0.0447) | (0.768) | (0.212) | (0.0757) | (0.0901) | (0.340) | (0.0820) | (0.0307) | (0.0498) |
| Pre-Fire Outcome (January-May 2015) | 1.032^***^ | 0.957^***^ | 0.751^***^ | 1.053^***^ | 0.890^***^ | 0.896^***^ | 0.669^***^ | 1.005^***^ | 1.163^***^ | 1.192^***^ | 0.927^***^ | 1.285^***^ |
|  | (0.0703) | (0.0249) | (0.0254) | (0.0325) | (0.0275) | (0.0303) | (0.0317) | (0.0412) | (0.128) | (0.0379) | (0.0375) | (0.0416) |
| Time Dummies | Y | Y | Y | Y | Y | Y | Y | Y | Y | Y | Y | Y |
| N: District-Month | 2,203 | 2,203 | 2,203 | 2,203 | 451 | 451 | 451 | 451 | 1,752 | 1,752 | 1,752 | 1,752 |

Standard errors in parentheses * p<0.10; ** p<0.05; *** p<0.01

Note: District JKN Member use January 2015 JKN member per district figure as a baseline.

### Appendix 8. The Effect of forest fire affected districts on hospital care utilization over five years old (urban vs rural) (ANCOVA Regression)

|  | City + Regency | | | | City (urban) | | | | Regency (rural) | | | |
| --- | --- | --- | --- | --- | --- | --- | --- | --- | --- | --- | --- | --- |
|  | (1) | (2) | (3) | (4) | (5) | (6) | (7) | (8) | (9) | (10) | (11) | (12) |
|  | Total Visit per 1,000 Under Five Years JKN members | Respiratory visits per 1,000 Under Five Years JKN members | Common Cold visit per 1,000 Under Five JKN Years members | ARTI visit per 1,000 Under Five Years JKN members | Total Visit per 1,000 Under Five Years JKN members | Respiratory visits per 1,000 Under Five Years JKN members | Common Cold visit per 1,000 Under Five JKN Years members | ARTI visit per 1,000 Under Five Years JKN members | Total Visit per 1,000 Under Five Years JKN members | Respiratory visits per 1,000 Under Five Years JKN members | Common Cold visit per 1,000 Under Five JKN Years members | ARTI visit per 1,000 Under Five Years JKN members |
| During Forest Fire | -0.200 | 0.00542 | -0.00289 | 0.00213 | 3.210^**^ | 0.102 | -0.00306 | 0.0146 | -1.245^***^ | -0.0240 | -0.00343 | 0.00152 |
|  | (0.396) | (0.0343) | (0.00301) | (0.00555) | (1.312) | (0.0985) | (0.00424) | (0.0131) | (0.353) | (0.0376) | (0.00367) | (0.00643) |
| After Forest Fire | 0.235 | 0.0377 | -0.00145 | 0.000518 | 5.211^***^ | -0.0687 | -0.00320 | -0.0231^**^ | -1.578^***^ | 0.0523 | -0.00197 | 0.00905 |
|  | (0.454) | (0.0394) | (0.00257) | (0.00630) | (1.455) | (0.113) | (0.00634) | (0.0105) | (0.417) | (0.0391) | (0.00283) | (0.00798) |
| Pre-Fire Outcome (January-May 2015) | 1.032^***^ | 0.737^***^ | 0.495^***^ | 0.853^***^ | 0.990^***^ | 0.697^***^ | 0.762^***^ | 0.559^***^ | 0.946^***^ | 0.743^***^ | 0.487^***^ | 0.952^***^ |
|  | (0.0125) | (0.0253) | (0.0579) | (0.108) | (0.0174) | (0.0327) | (0.0683) | (0.0421) | (0.0223) | (0.0479) | (0.0595) | (0.145) |
| Time Dummies | Y | Y | Y | Y | Y | Y | Y | Y | Y | Y | Y | Y |
| N: District-Month | 2,203 | 2,203 | 2,203 | 2,203 | 451 | 451 | 451 | 451 | 1,752 | 1,752 | 1,752 | 1,752 |

Standard errors in parentheses * p<0.10; ** p<0.05; *** p<0.01

Note: District JKN Member use January 2015 JKN member per district figure as a baseline.

### Appendix 9. Robustness Test for ANCOVA with Different AOD Threshold

**Primary Care Service**

|  |  | Under Five Years Old | | | | Over Five Years Old | | | |
| --- | --- | --- | --- | --- | --- | --- | --- | --- | --- |
|  |  | (1) | (2) | (3) | (4) | (5) | (6) | (7) | (8) |
|  |  | Total Visit per 1,000 Under Five members | Respiratory visits per 1,000 Under Five members | Common Cold visit per 1,000 Under Five members | ARTI visit per 1,000 Under Five members | Total Visit per 1,000 Over Five members | Respiratory visits per 1,000 Over Five members | Common Cold visit per 1,000 Over Five members | ARTI visit per 1,000 Over Five members |
| **Threshold: 0.50**  **Treated=184; Control=36** | During Forest Fire | 5.937^**^ | 2.876^*^ | 1.435 | 0.852^***^ | 0.614^*^ | 0.154^*^ | -0.110^**^ | 0.111^***^ |
|  |  | (2.328) | (1.571) | (0.970) | (0.213) | (0.314) | (0.0848) | (0.0458) | (0.0239) |
|  | After Forest Fire | 7.667^***^ | 5.123^***^ | 3.182^***^ | 0.650 | 1.327^***^ | 0.283^**^ | -0.107^**^ | 0.134^***^ |
|  |  | (2.488) | (1.775) | (1.085) | (0.409) | (0.449) | (0.112) | (0.0434) | (0.0448) |
| **Threshold: 0.75** | During Forest Fire | 1.421 | 0.432 | 0.264 | -0.277 | 0.160 | 0.0312 | -0.0135 | 0.00239 |
| **Treated= 89; Control=131** |  | (1.408) | (1.089) | (0.791) | (0.269) | (0.259) | (0.0606) | (0.0258) | (0.0295) |
|  | After Forest Fire | 3.130^*^ | 2.406^*^ | 2.136^***^ | -0.655 | 0.312 | 0.0679 | -0.0845^***^ | 0.0661 |
|  |  | (1.634) | (1.251) | (0.801) | (0.406) | (0.347) | (0.0822) | (0.0290) | (0.0447) |

Note: Standard errors in parentheses ^*^ *p* < 0.10, ^**^ *p* < 0.05, ^***^ *p* < 0.01; Pre-fire outcome is not shown because do not change much. we use time dummies. N: District-Month equals to 2,203.

### Appendix 10. Robustness Test for ANCOVA with Different AOD Threshold

**Hospital Care Service**

|  |  | Under Five Years Old | | | | Over Five Years Old | | | |
| --- | --- | --- | --- | --- | --- | --- | --- | --- | --- |
|  |  | (1) | (2) | (3) | (4) | (5) | (6) | (7) | (8) |
|  |  | Total Visit per 1,000 Under Five members | Respiratory visits per 1,000 Under Five members | Common Cold visit per 1,000 Under Five members | ARTI visit per 1,000 Under Five members | Total Visit per 1,000 Over Five members | Respiratory visits per 1,000 Over Five members | Common Cold visit per 1,000 Over Five members | ARTI visit per 1,000 Over Five members |
| **Threshold: 0.50**  **Treated=184; Control=36** | During Forest Fire | -5.175^***^ | -2.024^***^ | -0.262^*^ | -0.291 | -0.512 | -0.0963 | -0.0123^**^ | -0.0140 |
|  |  | (1.571) | (0.630) | (0.157) | (0.200) | (0.604) | (0.0632) | (0.00581) | (0.0100) |
|  | After Forest Fire | -7.209^***^ | -1.791^***^ | 0.113 | -0.297 | -1.029 | 0.00302 | -0.00677 | -0.000142 |
|  |  | (1.697) | (0.535) | (0.128) | (0.222) | (0.653) | (0.0655) | (0.00438) | (0.00975) |
| **Threshold: 0.75** | During Forest Fire | -3.261^***^ | -0.275 | -0.132 | 0.170 | -0.200 | 0.00542 | -0.00289 | 0.00213 |
| **Treated= 89; Control=131** |  | (0.951) | (0.318) | (0.0812) | (0.105) | (0.396) | (0.0343) | (0.00301) | (0.00555) |
|  | After Forest Fire | -5.147^***^ | -0.0767 | 0.141^*^ | 0.0606 | 0.235 | 0.0377 | -0.00145 | 0.000518 |
|  |  | (1.244) | (0.298) | (0.0791) | (0.127) | (0.454) | (0.0394) | (0.00257) | (0.00630) |

Note: Standard errors in parentheses ^*^ *p* < 0.10, ^**^ *p* < 0.05, ^***^ *p* < 0.01; Pre-fire outcome is not shown because do not change much. We use time dummies. N: District-Month equals to 2,203.

Generally, examining the robustness strengthens rather than weakens our results. For instance, comparing two cutoff applications (0.50 vs 0.75), we observe that all primary care visits in children under five years old during the fire period yield estimates four times larger (5.94 vs 1,42) and twofold higher after the fire (7.67 vs 3.13). Similarly, respiratory disease and ARTI visits also show higher effect estimates during and after the forest fire. This pattern is also evident in individuals over five years old, where initially nonsignificant estimates become significant. The estimates for hospital care are now significant but lower compared to those for primary care services for the under five years age group, with greater forgone care in hospital services during (-3.26 vs -5.18) and after the forest fire (-5.15 vs -7.21). Furthermore, we now find significant negative effects for visits to the hospital for respiratory diseases (-2.02) and common colds (-0.26) in the same age group. The changes in estimates after introducing a lower cutoff are less apparent in hospital care visits for individuals over five years old, with only common cold visits showing a significant negative effect (-0.012).

**Appendix 11A. Ordinary Least Square Regression with forest fire affected status, month dummies, and interaction of time dummies and treatment status (Primary Care)**

|  | (1) | (2) | (3) | (4) | (5) | (6) | (7) | (8) |
| --- | --- | --- | --- | --- | --- | --- | --- | --- |
|  | Total Visit per 1,000 Under Five members | Respiratory visits per 1,000 Under Five members | Common Cold visit per 1,000 Under Five members | ARTI visit per 1,000 Under Five members | Total Visit per 1,000 Over Five members | Respiratory visits per 1,000 Over Five members | Common Cold visit per 1,000 Over Five members | ARTI visit per 1,000 Over Five members |
| Affected Districts (1=Yes; 0=No) | 1.047 | 0.628 | 0.251 | 0.512 | -1.671 | -0.0199 | -0.126 | 0.114 |
|  | (6.535) | (3.891) | (3.539) | (0.997) | (1.398) | (0.310) | (0.114) | (0.135) |
| February 2015 | 1.142 | 0.911 | 0.0901 | 0.593 | -0.346 | 0.0533 | 0.00156 | 0.0111 |
|  | (7.117) | (4.238) | (3.854) | (1.086) | (1.523) | (0.337) | (0.124) | (0.147) |
| March 2015 | 3.881 | 2.982 | 1.150 | 1.394 | -0.601 | 0.246 | 0.0743 | 0.0936 |
|  | (7.161) | (4.264) | (3.878) | (1.092) | (1.533) | (0.339) | (0.125) | (0.147) |
| April 2015 | 4.053 | 2.574 | 0.967 | 1.234 | -0.387 | 0.184 | 0.0294 | 0.0487 |
|  | (7.161) | (4.264) | (3.878) | (1.092) | (1.533) | (0.339) | (0.125) | (0.147) |
| May 2015 | 2.839 | 1.784 | 0.484 | 1.151 | 0.623 | 0.163 | -0.00378 | 0.0657 |
|  | (7.139) | (4.251) | (3.866) | (1.089) | (1.528) | (0.338) | (0.125) | (0.147) |
| June 2015 | 2.824 | 1.698 | 0.0920 | 1.213 | -0.310 | 0.156 | -0.0272 | 0.0757 |
|  | (7.139) | (4.251) | (3.866) | (1.089) | (1.528) | (0.338) | (0.125) | (0.147) |
| 1.Affected#February 2015 | 1.511 | 1.935 | 2.022 | -0.185 | 0.545 | -0.0228 | 0.0115 | -0.0237 |
|  | (9.241) | (5.502) | (5.004) | (1.410) | (1.978) | (0.438) | (0.161) | (0.190) |
| 1.Affected#March 2015 | 2.163 | 0.424 | 0.886 | -0.746 | 1.877 | -0.120 | -0.0323 | -0.0730 |
|  | (9.283) | (5.527) | (5.027) | (1.416) | (1.987) | (0.440) | (0.162) | (0.191) |
| 1.Affected#April 2015 | 3.039 | 1.561 | 1.998 | -0.878 | 1.779 | -0.104 | -0.0436 | -0.0392 |
|  | (9.283) | (5.527) | (5.027) | (1.416) | (1.987) | (0.440) | (0.162) | (0.191) |
| 1.Affected#May 2015 | 1.389 | 1.626 | 2.046 | -0.801 | -0.136 | -0.174 | -0.0115 | -0.0900 |
|  | (9.258) | (5.512) | (5.013) | (1.412) | (1.981) | (0.439) | (0.162) | (0.191) |
| 1.Affected#June 2015 | 4.671 | 2.071 | 2.731 | -0.818 | 1.187 | -0.131 | 0.0256 | -0.0860 |
|  | (9.266) | (5.517) | (5.018) | (1.413) | (1.983) | (0.439) | (0.162) | (0.191) |
| Constant | 13.56^***^ | 7.537^**^ | 2.799 | 2.460^***^ | 7.044^***^ | 1.334^***^ | 0.465^***^ | 0.363^***^ |
|  | (5.033) | (2.996) | (2.725) | (0.768) | (1.077) | (0.238) | (0.0878) | (0.104) |
| *N* | 1,215 | 1,215 | 1,215 | 1,215 | 1,215 | 1,215 | 1,215 | 1,215 |

**Appendix 11B. Ordinary Least Square Regression with forest fire affected status, month dummies, and interaction of time dummies and treatment status (Hospital Care)**

|  | (1) | (2) | (3) | (4) | (5) | (6) | (7) | (8) |
| --- | --- | --- | --- | --- | --- | --- | --- | --- |
|  | Total Visit per 1,000 Under Five members | Respiratory visits per 1,000 Under Five members | Common Cold visit per 1,000 Under Five members | ARTI visit per 1,000 Under Five members | Total Visit per 1,000 Over Five members | Respiratory visits per 1,000 Over Five members | Common Cold visit per 1,000 Over Five members | ARTI visit per 1,000 Over Five members |
| Affected District (1=Yes; 0=No) | -1.621 | -1.171 | -0.00576 | -0.328 | 1.208 | -0.166 | -0.0202 | 0.00575 |
|  | (4.987) | (1.135) | (0.285) | (0.386) | (3.910) | (0.232) | (0.0176) | (0.0212) |
| February 2015 | 1.630 | 1.173 | 0.145 | 0.223 | -0.249 | 0.0455 | -0.0144 | 0.0212 |
|  | (5.432) | (1.237) | (0.311) | (0.420) | (4.259) | (0.253) | (0.0191) | (0.0231) |
| March 2015 | 12.31^**^ | 3.207^**^ | 0.262 | 0.324 | 4.292 | 0.205 | -0.0155 | -0.00122 |
|  | (5.465) | (1.244) | (0.313) | (0.423) | (4.285) | (0.254) | (0.0192) | (0.0233) |
| April 2015 | 9.094^*^ | 1.838 | 0.174 | -0.123 | 4.272 | 0.115 | -0.0130 | -0.0179 |
|  | (5.465) | (1.244) | (0.313) | (0.423) | (4.285) | (0.254) | (0.0192) | (0.0233) |
| May 2015 | 6.950 | 1.058 | 0.165 | -0.257 | 1.899 | -0.0149 | -0.0221 | -0.0171 |
|  | (5.448) | (1.240) | (0.312) | (0.422) | (4.272) | (0.253) | (0.0192) | (0.0232) |
| June 2015 | 6.942 | -0.0207 | 0.102 | -0.841^**^ | 2.309 | -0.0805 | -0.0226 | -0.0307 |
|  | (5.448) | (1.240) | (0.312) | (0.422) | (4.272) | (0.253) | (0.0192) | (0.0232) |
| 1.Affected#February 2015 | -0.906 | -0.836 | -0.183 | -0.181 | -0.0107 | 0.0122 | 0.0336 | -0.0121 |
|  | (7.053) | (1.606) | (0.403) | (0.546) | (5.530) | (0.328) | (0.0248) | (0.0300) |
| 1.Affected#March 2015 | -6.149 | -1.567 | -0.328 | -0.108 | -1.966 | -0.212 | 0.0147 | -0.00185 |
|  | (7.084) | (1.613) | (0.405) | (0.548) | (5.555) | (0.329) | (0.0249) | (0.0302) |
| 1.Affected#April 2015 | -5.028 | -1.524 | -0.333 | 0.0244 | -2.720 | -0.191 | 0.00285 | -0.00629 |
|  | (7.084) | (1.613) | (0.405) | (0.548) | (5.555) | (0.329) | (0.0249) | (0.0302) |
| 1.Affected#May 2015 | -5.307 | -2.130 | -0.476 | -0.233 | -1.643 | -0.135 | 0.0132 | 0.000921 |
|  | (7.065) | (1.609) | (0.404) | (0.547) | (5.540) | (0.328) | (0.0249) | (0.0301) |
| 1.Affected#June 2015 | -4.668 | -1.068 | -0.355 | 0.299 | -0.994 | -0.105 | 0.0103 | -0.00156 |
|  | (7.071) | (1.610) | (0.405) | (0.547) | (5.545) | (0.329) | (0.0249) | (0.0301) |
| Constant | 32.08^***^ | 6.959^***^ | 0.819^***^ | 2.125^***^ | 20.53^***^ | 1.091^***^ | 0.0494^***^ | 0.0880^***^ |
|  | (3.841) | (0.874) | (0.220) | (0.297) | (3.012) | (0.179) | (0.0135) | (0.0163) |
| *N* | 1,215 | 1,215 | 1,215 | 1,215 | 1,215 | 1,215 | 1,215 | 1,215 |

**Appendix 12. F-Test Report using Ordinary Least Square Regression with time, forest fire affected status, and interaction of time dummies and treatment status (Primary Care and Hospital Care)**

|  |  |  | January-June 2015 (6 month) | Reject Ho or Not |
| --- | --- | --- | --- | --- |
| Type of Facility | Age Group | Outcome | Interaction of time dummies and district treatment status |  |
| Primary Care | Under Five Years Old | All Visits | Prob > F = 0.9977 | No |
|  |  | Respiratory Disease | Prob > F = 0.9986 | No |
|  |  | Common Cold | Prob > F = 0.9956 | No |
|  |  | ARTI | Prob > F = 0.9821 | No |
|  | Over Five Years Old | All Visits | Prob > F = 0.8588 | No |
|  |  | Respiratory Disease | Prob > F = 0.9987 | No |
|  |  | Common Cold | Prob > F = 0.9983 | No |
|  |  | ARTI | Prob > F = 0.9961 | No |
| Hospital Care | Under Five Years Old | All Visits | Prob > F = 0.9337 | No |
|  |  | Respiratory Disease | Prob > F = 0.8377 | No |
|  |  | Common Cold | Prob > F = 0.8916 | No |
|  |  | ARTI | Prob > F = 0.9437 | No |
|  | Over Five Years Old | All Visits | Prob > F = 0.9956 | No |
|  |  | Respiratory Disease | Prob > F = 0.9756 | No |
|  |  | Common Cold | Prob > F = 0.8079 | No |
|  |  | ARTI | Prob > F = 0.9982 | No |

Note: Number of district-month observations are 1,215.

**References**

1. GIS-Resources. Interpolation - GIS Resources [Internet]. 2013 [cited 2021 Oct 27]. Available from: https://gisresources.com/gis_interpolation_techniques_2/

2. Mitas, Mitasova. Spatial interpolation. P.Longley MFGDJMDWR (Eds. ), editor. Geographical Information Systems: Principles, Techniques, Management and Applications, GeoInformation International. Wiley; 1999.

3. Chen CC, Wang YR, Yeh HY, Lin TH, Huang CS, Wu CF. Estimating monthly PM2.5 concentrations from satellite remote sensing data, meteorological variables, and land use data using ensemble statistical modeling and a random forest approach. Environmental Pollution. 2021 Dec 15;291.
